# Supplementary material for: Theory predicts 2D chiral polaritons based on achiral Fabry–Pérot cavities using apparent circular dichroism
Source: Nat Commun. 2024 Jan 6;15:340. doi: 10.1038/s41467-023-44523-1 (PMC10771534; doi:10.1038/s41467-023-44523-1)
Supplement: Supplementary file 1 — Supplementary Information [file 41467_2023_44523_MOESM1_ESM.pdf]

Supplementary information for "Theory predicts 2D chiral polaritons based on achiral Fabry–Pérot cavities using apparent circular dichroism"

Andrew H. Salij,[†] Randall H. Goldsmith,[‡] and Roel Tempelaar[*,†]

†*Department of Chemistry, Northwestern University, 2145 Sheridan Road, Evanston, Illinois 60208, USA*

‡*Department of Chemistry, University of Wisconsin-Madison, Madison, WI 53706-1322, USA*

E-mail: roel.tempelaar@northwestern.edu

Contents

Supplementary Note 1: Conventions **S2**

Supplementary Note 2: Software **S2**

Supplementary Methods 1: Derivations **S2**

Interaction Hamiltonian S2

Apparent circular dichroism S5

Polaritonic characteristics S8

Small path length limit of the polarizance parameters S9

Supplementary Methods 2: Mirrors as reciprocal boundaries **S10**

Supplementary Figure 1: Inversion of inter-dipole angle **S12**

Supplementary References **S12**

Supplementary Note 1: Conventions

This paper reports on interdisciplinary work spanning chemistry, classical optics, and quantum optics, which differ in their conventions with regards to circular polarizations and to circular dichroism. Bearing this in mind, we keep with a convention standard to Mueller calculus, considering polarization from the perspective of the receiver. This means that the electric field of right-handed circular polarization (r) can be expressed as $\mathbf{E}_r = \mathbf{E}_x + i\mathbf{E}_y$ and the complex conjugate of this for left-handed circular polarization (l).

Generally, chiroptical response is defined as l minus r, which is the opposite of the intensities of the element of the Stokes vector corresponding to circular polarization (s_3). Here, we invoke the latter for the Mueller calculus, as doing so maintains a consistent treatment of sign for all forms of dichroism demonstrating Lorentz group symmetries, but the direct handling of apparent circular dichroism (ACD) and the main text switches to the former for consistency with the main body of spectroscopy literature.

Supplementary Note 2: Software

Software written in Python 3.8 using numpy (1.22.3),[1] scipy (1.7.3),[2] and matplotlib (3.5.1).[3] Vector graphics created using Inkscape and Powerpoint (16.79).

Supplementary Methods 1: Derivations

Interaction Hamiltonian

Let us first consider the case of normal absorption in the Coulomb gauge. Omitting the dipole self-energy, the classical light–matter interaction Hamiltonian of a single electron is[4]

$$H_{\text{int}} = -\frac{e}{m_e}(\mathbf{A}(r,t)\cdot\mathbf{p}), \tag{S1}$$

where **A** is the light vector potential, which we assume takes the plane-wave form

$$\mathbf{A}(r,t) = \mathbf{A}_0(e^{-i\phi} + e^{i\phi}) \tag{S2}$$

$$\phi = \omega t - kr$$

$$\mathbf{A}_0 = \begin{pmatrix} A_{0,x} \\ A_{0,y} \end{pmatrix}.$$

We will next take the long-wavelength approximation of $\mathbf{A}_0 e^{\pm i\phi} = \mathbf{A}_0(1 \mp ikr \pm ...)$ and will only consider the first term, the electric field-electric dipole interaction. Without loss of generality, we will only directly evaluate the contribution from $A_{0,x}$ as the analysis is identical for the other polarization term,

$$H_{\text{int}} = -\frac{e}{m_e} A_{0,x} p_x (e^{i\phi} + e^{-i\phi}). \tag{S3}$$

From here, we will only directly consider the first term, knowing that the latter is simply its complex conjugate. Furthermore, we will now directly consider the momentum as an operator $\hat{p}$. Since, for an electron e, the Heisenberg equation of motion implies that $\hat{p}_x = \frac{im_e}{\hbar}[\hat{H}_e, \hat{r}_x]$, the action of $\hat{p}_x$ transitioning from the electronic ground state $|0\rangle$ to excited state $|n\rangle$ is given as

$$\langle n| \hat{p}_x |0\rangle = \frac{im_e}{\hbar} \langle n| \hat{H}_e \hat{r}_x - \hat{r}_x \hat{H}_e |0\rangle = \frac{im_e}{\hbar}(E_n - E_0) \langle n| \hat{r}_x |0\rangle = im_e \omega_n \langle n| \hat{r}_x |0\rangle . \tag{S4}$$

Considering all polarizations, all excited-state transitions indexed by n, and both counter-rotating terms of the vector potential, the light–matter interaction Hamiltonian may be re-expressed as[4]

$$H_{\text{int}} = \sum_n -i\omega_n \mathbf{A} \cdot \boldsymbol{\mu}_n, \tag{S5}$$

where $\boldsymbol{\mu} = e\mathbf{r}$, which is the electric dipole interaction in the same basis as the vector potential. Here, we write $\boldsymbol{\mu}_n$ to refer specifically to the transition dipole from the ground state to state n as we have summed the interaction over multiple excited states. Omitting such indexing and as it is the electric dipole interaction with the electric field, this interaction is often written as $-\mathbf{E} \cdot \boldsymbol{\mu}$. For the case of a crystal, the dielectric tensor

dressed by exciton states may also be expressed in terms of dipole projections on to the tensor axes, namely

$$\epsilon_{ij}(\omega) = \delta_{ij} + \frac{2}{\hbar\epsilon_0 v}\sum_n \frac{\mu_{n,i}\mu_{n,j}}{\omega_n^2 - \omega^2}\omega_n \tag{S6}$$

at $\mathbf{k} = \mathbf{0}$ in the case of negligible spatial dispersion and where v is the unit cell volume.[5] Adding to this expression, we make two alterations to account for isotropic high-frequency contributions to the dielectric given by ϵ_∞ and to account for damping,[5,6]

$$\epsilon_{ij}(\omega) = \epsilon_\infty\delta_{ij} + \frac{2}{\hbar\epsilon_0 v}\sum_n \frac{\mu_{n,i}\mu_{n,j}}{\omega_n^2 - \omega^2 - i\gamma_n\omega}\omega_n. \tag{S7}$$

Along a transition axis, the dielectric contribution of a single exciton state to the *imaginary* component of the dielectric diagonal component along that axis is found to be

$$\epsilon_{i,n} = \frac{2\mu_n^2}{\epsilon_0\hbar v}\frac{\omega_n\omega\gamma_n}{(\omega_n^2 - \omega^2)^2 + \gamma_n^2\omega^2}. \tag{S8}$$

In principle, this formulation of the dielectric should agree with one directly arising from Fermi's Golden Rule. That is, we can relate the transition rate Γ_n to a characteristic absorption $\bar{\alpha} = \bar{A}/l$, in turn related to a natural absorbance $\bar{A}$,[7] which was also related to the imaginary dielectric,

$$\frac{\omega\epsilon_{i,n}}{nc} = \bar{\alpha} = \frac{\hbar\omega\Gamma_n}{|\mathbf{S}|}, \tag{S9}$$

where $\mathbf{S} = \frac{1}{2\mu_0}(\mathbf{E}\times\mathbf{B})$ is the time-averaged Poynting vector. For the case of the plane wave, we will re-express said vector in terms of the vector potential in order to find the magnitude. That is, $\mathbf{B} = \nabla\times\mathbf{A}$ and $\mathbf{E} = -\frac{d}{dt}\mathbf{A}$. For notational convenience, we will refer to $|A_0|$, the absolute value of the vector potential, predominantly from now on and work with norms of complex quantities. Upon rearrangement and determination that $|\mathbf{S}| = \frac{\omega^2 n|A_0|^2}{2\mu_0 c}$ for non-magnetic materials, we find that[8]

$$\epsilon_{i,n} = \frac{2\hbar\Gamma_n}{\omega^2\epsilon_0|A_0|^2}. \tag{S10}$$

The transition rate of a given exciton transition is given as $\Gamma_n = \frac{2\pi}{\hbar}|H_{\text{int},n}|^2\rho(\hbar\omega)$, with ρ here being the density of states due to damping. For the case of normal absorption, the light–matter interaction for a

specific transition n, $H_{\text{int},n}$, is already bounded, namely

$$|H_{\text{int},n}| = \omega_n \mu_n |A_0|. \tag{S11}$$

From comparison of the two forms of the dielectric, one obtains

$$|A_0|^2 \omega_n^2 \mu_n^2 \rho(\hbar\omega) = |A_0|^2 \mu_n^2 \frac{\omega^2}{2\pi\hbar v} \frac{\omega_n \omega \gamma_n}{(\omega_n^2 - \omega^2)^2 + \gamma_n^2 \omega^2}, \tag{S12}$$

which implies

$$\rho(\hbar\omega) = \frac{1}{2\pi\hbar v} \frac{\omega^2}{\omega_n^2} \frac{\omega_n \omega \gamma_n}{((\omega_n^2 - \omega^2)^2 + \gamma_n^2 \omega^2)}. \tag{S13}$$

In effect, we are accounting for the broadening due to damping, which produces a Lorentzian density of states,[5,9] and thereby creates an effective width in line with previous polaritonic studies.[10] For this analysis, we will focus on purely electronic optical transitions and reintroduce lineshapes where necessary.

Apparent circular dichroism

For the case of ACD, we will be primarily considering characteristic absorption lengths. By definition, we have

$$\alpha_{l/r} = \alpha_{avg} \pm \alpha_{CD}. \tag{S14}$$

We should discuss briefly how these absorption lengths relate to the Mueller parameters. For a macroscopic matrix of the form $\mathbf{M} = e^{-\bar{\alpha} z}\mathbf{m}$, the inverse lengths are:

$$\alpha_{avg} = \bar{\alpha} - \frac{1}{l}\log(m_{00}) \tag{S15}$$

$$\alpha_{l/r} = \bar{\alpha} - \frac{1}{l}\log(m_{00} \pm m_{03}). \tag{S16}$$

As we are working in the case of relatively small pathlength, we introduce the following approximations to render the problem analytically tractable. For sufficiently small $|\beta|$ and $|\mathbf{d}|$ compared to mean absorption, $m_{00} \approx 1$ and $\log(m_{00} \pm m_{03}) \approx m_{00} - 1 \pm m_{03} \approx \pm m_{03}$, enabling conflation of matrix elements with absorptions

as

$$\alpha_{avg} = \bar{\alpha} \tag{S17}$$

$$\alpha_{CD} = \frac{\alpha_l - \alpha_r}{2} = m_{03}/l. \tag{S18}$$

This conflation is implied in standard second-order treatments of the Mueller matrix [11,12] and is necessary to have a compact mathematical framework. Under these bounds and from previous analysis, [13] we know that

$$\bar{\alpha}_n(\omega) = \frac{\omega \epsilon_{i,n}(\omega)}{n(\omega)c} = \omega \xi \mu_n^2 \omega_n V_n(\omega) \tag{S19}$$

$$\alpha_{CD,n} \approx \sum_{m,n} \frac{B_1}{l} \omega^2 \xi^2 \mu_m^2 \omega_m W_m(\omega) \mu_n^2 \omega_n V_n(\omega) \sin(2\beta_{mn}), \tag{S20}$$

where $\xi = \frac{1}{\hbar c \epsilon_0 \sqrt{\epsilon_\infty} v}$ under the perturbative case. Here, $n(\omega) \approx \sqrt{\epsilon_\infty}$, $\beta_{mn} \equiv \beta_m - \beta_n$, where β_i is the ith dipole angle from the x-axis, and B_1 is the first of Brown's polarizance parameters. [14] Therefore, circular absorption is given as

$$\alpha_{l/r,n} = \omega \xi \mu_n^2 \omega_n V_n \Big(1 \pm \sum_m \frac{B_1}{l} \omega \xi \mu_m^2 \omega_m W_m \sin(2\beta_{mn})\Big) \tag{S21}$$

$$\alpha_{l/r,n} = 2\omega \xi \mu_n^2 \omega_n V_n (\frac{1}{2} \pm \frac{1}{2}\sigma_n) \tag{S22}$$

$$\sigma_n = \sum_m \frac{B_1}{l} \omega \xi \mu_m^2 \omega_m W_m \sin(2\beta_{mn}), \tag{S23}$$

with σ_n being the chiral interaction term. Relating this expression to an effective Fermi's Golden Rule-type expression as in the previous subsection, we have

$$\alpha_{l/r,n} = 2\omega \xi \mu_n^2 \omega_n V_n (\frac{1}{2} \pm \frac{1}{2}\sigma_n) = 2\frac{2\pi \mu_0 c}{\omega n(\omega)|A_0|^2} |H_{\text{int},n,l/r}|^2 \rho(\hbar\omega), \tag{S24}$$

where we have separated the interaction Hamiltonian of state n over the polarization basis as $H_{\text{int},n,l/r}$. Treating $n(\omega) \approx \sqrt{\epsilon_\infty}$, the above expression rearranges to

$$\omega^2 |A_0|^2 \mu_n^2 \omega_n V_n (\frac{1}{2} \pm \frac{1}{2}\sigma_n) = 2\pi \hbar v |H_{\text{int},n,l/r}|^2 \rho(\hbar\omega). \tag{S25}$$

Reintroducing $\rho(\hbar\omega) = \frac{1}{2\pi\hbar v}\frac{\omega^2}{\omega_n}V_n$ from (S13), the above simplifies to

$$\omega_n^2|A_0|^2|\mu_n|^2(\frac{1}{2} \pm \frac{1}{2}\sigma_n) = |H_{\text{int},n,l/r}|^2, \tag{S26}$$

giving

$$|A_0|\omega_n\mu_n\sqrt{\frac{1}{2} \pm \frac{1}{2}\sum_m \frac{B_1}{l}\omega\xi\mu_m^2\omega_m W_m \sin(2\beta_{mn})} = |H_{\text{int},n,l/r}|. \tag{S27}$$

Note that in the case of linear absorption, this reduces to the same form as in the previous subsection but halved for each circular mode.

To return to the interaction Hamiltonian as a quantum mechanical operator, the vector potential should be considered as an operator as well. In the general case of a quantized electromagnetic field, the vector potential may be discretized into photonic creation and annihilation operators in terms of the mode volume V_0 as

$$\mathbf{A} \rightarrow \sum_{\lambda=\pm}\sqrt{\frac{\hbar}{2\epsilon_0\omega V_0}}\mathbf{e}_\lambda\{\hat{a}_\lambda e^{-i\omega t} + \hat{a}_\lambda^\dagger e^{i\omega t}\}, \tag{S28}$$

with the index plus(minus) labelled by λ referring to the polarization modes discussed in the main text, and where we have asserted that $\mathbf{k}\cdot\mathbf{r} = 0$ in the regime of interest. To further clarify, $\mathbf{e}_\lambda$ is the λ polarization component of the unit vector of $\mathbf{A}$ in the $\pm$ basis. For an interaction Hamiltonian acting on a collection of dipole two-level systems, one has, under the dipole and rotating-wave approximations,[15,16]

$$\hat{H}_{\text{int}} = i\sum_n\sum_{\lambda=\pm} A_{0,\lambda}\omega_n\tilde{\mu}_{n,\lambda}(\hat{a}_\lambda^\dagger\hat{b}_n - \hat{a}_\lambda\hat{b}_n^\dagger), \tag{2}$$

with $A_{0,\lambda}$ being the absolute value of the component in direction λ of $\mathbf{A}$. That is, $A_{0,\lambda} = \sqrt{\frac{\hbar}{2\epsilon_0\omega_\lambda V_0}}\mathbf{e}_\lambda$. For the case of ACD, we will introduce the indexing variable $\tau_\lambda = \pm 1$ which is positive/negative for left-handed/right-handed circular polarization, respectively. This allows a full discretization of the Hamiltonian

as

$$\hat{H}_{\mathrm{int}}(l) = i \sum_{n} \sum_{\lambda=\pm} A_{0,\lambda} \omega_n \tilde{\mu}_n (\hat{a}_{\lambda}^{\dagger} \hat{b}_n - \hat{a}_{\lambda} \hat{b}_n^{\dagger}) \sqrt{\frac{1}{2} + \frac{1}{2} \tau_{\lambda} \sigma_n}, \tag{S29}$$

with

$$\sigma_n = \sum_{m \neq n} \frac{B_1}{l} \Omega \xi \mu_m^2 \omega_m W_m(\Omega) \sin(2\beta_{mn}). \tag{S30}$$

This expression is what we use to numerically calculate the chiral interaction in the polaritonic calculations. Taking the approximation $\frac{B_1}{l} \approx \frac{1}{2} l^2$ for the short pathlength case, we have

$$\sigma_n \equiv \frac{1}{2} l \xi \omega \sum_{m \neq n} \mu_m^2 \omega_m W_m(\omega) \sin(2\beta_{mn}), \tag{8}$$

as discussed in the main text.

Polaritonic characteristics

From the main text, the polaritonic mixing for a given eigenstate α is given by

$$\chi^{\alpha} = 2|C_e^{\alpha} C_{\gamma}^{\alpha}|, \tag{S31}$$

where C_e^{α} is the coefficient for all excited states and C_{γ}^{α} that for all photonic states. Here, we will derive this within the three state approximation.

For the two polaritons associated with electronic state n, the coefficients are

$$C_{\gamma,(n)}^{u/l} = \cos(\tan^{-1}(\Pi_{(n)}^{u/l}))\Pi_{(n)}^{u/l} \tag{S32}$$

$$C_{e,(n)}^{u/l} = \cos(\tan^{-1}(\Pi_{(n)}^{u/l})). \tag{S33}$$

Via substitution, one finds that the polaritonic mixing is given by the following:

$$\begin{aligned}\chi_{(n)}^{u/l} &= 2|\cos^2(\tan^{-1}(\Pi_{(n)}^{u/l}))\Pi_{(n)}^{u/l}| \\ &= 2\left|\frac{\Pi_{(n)}^{u/l}}{\left(\Pi_{(n)}^{u/l}\right)^2 + 1}\right|.\end{aligned} \tag{S34}$$

Substituting $\Pi_{(n)}^{u/l} = \frac{\Phi_n}{\Delta_n \pm \sqrt{\Delta_n^2 + \Phi_n^2}}$ and algebraic manipulation yields the simplified expression

$$\chi_{(n)}^{u/l} = \frac{\Phi_n}{\sqrt{\Delta_n^2 + \Phi_n^2}}. \tag{24}$$

The dissymmetry factor is given by a normalized difference of circularly-polarized coefficients, namely

$$g^{\alpha} = 2\frac{|C_l^{\alpha}|^2 - |C_r^{\alpha}|^2}{|C_l^{\alpha}|^2 + |C_r^{\alpha}|^2}. \tag{S35}$$

Omitting common factors, which cancel out, for both polaritons

$$g_{(n)}^{u/l} = 2\frac{(1+\sigma_n) - (1-\sigma_n)}{(1+\sigma_n) + (1-\sigma)} = 2\sigma_n. \tag{23}$$

Small path length limit of the polarizance parameters

From Eq. 30 presented in the Methods section of the main text, the small path length limit of B_0 and B_1 are readily evaluated through a Taylor expansion in l, yielding

$$\begin{aligned}B_0 &= \frac{R^2}{N^2}\left(1 + \frac{I^2 l^2}{2}\right) + \frac{I^2}{N^2}\left(1 - \frac{R^2 l^2}{2}\right) + O\left(l^4\right) \\ &= \frac{R^2 + I^2}{N^2} + O\left(l^4\right) \\ &\approx 1.\end{aligned} \tag{S36}$$

and

$$
\begin{aligned}
B_1 &= \frac{1}{N^2}\left\{1 + \frac{I^2 l^2}{2} - \left(1 - \frac{R^2 l^2}{2}\right)\right\} + O\left(l^4\right) \\
&= \frac{I^2 + R^2}{2N^2} l^2 + O\left(l^4\right) \\
&\approx \frac{1}{2} l^2,
\end{aligned}
\tag{S37}
$$

respectively.

Supplementary Methods 2: Mirrors as reciprocal boundaries

The Mueller calculus formalism underlying the quantum electrodynamical theory of ACD considers cavity mirrors as reciprocal boundaries imposed on the sample, which is justified in the following. Importantly, this treatment builds on the inversion antisymmetry of ACD, and ignores polarization effects of the mirrors.

Where there is a total absence of optical activity producing “genuine” circular dichroism, the differential Mueller matrix for forwards propagation in absence of mean absorption is

$$
\mathbf{H}_{\mathrm{F}} = \begin{pmatrix} 0 & d_1 & d_2 & 0 \\ d_1 & 0 & 0 & \beta_2 \\ d_2 & 0 & 0 & -\beta_1 \\ 0 & -\beta_2 & \beta_1 & 0 \end{pmatrix}. \tag{S38}
$$

For backwards propagation, the $x' - y'$ axes are interchanged in sign, implying that the backwards differential matrix is

$$
\mathbf{H}_{\mathrm{B}} = \begin{pmatrix} 0 & d_1 & -d_2 & 0 \\ d_1 & 0 & 0 & -\beta_2 \\ -d_2 & 0 & 0 & -\beta_1 \\ 0 & \beta_2 & \beta_1 & 0 \end{pmatrix}. \tag{S39}
$$

The macroscopic matrix for forwards propagation is

$$\mathbf{m}_\mathrm{F} = \begin{pmatrix} B_0 + B_1\left(d_1^2 + d_2^2\right) & -B_2 d_1 + B_3\beta_1 & -B_2 d_2 + B_3\beta_2 & B_1\left(-\beta_1 d_2 + \beta_2 d_1\right) \\ -B_2 d_1 + B_3\beta_1 & B_0 + B_1\left(-\beta_2^2 + d_1^2\right) & B_1\left(\beta_1\beta_2 + d_1 d_2\right) & -B_2\beta_2 - B_3 d_2 \\ -B_2 d_2 + B_3\beta_2 & B_1\left(\beta_1\beta_2 + d_1 d_2\right) & B_0 + B_1\left(-\beta_1^2 + d_2^2\right) & B_2\beta_1 + B_3 d_1 \\ -B_1\left(-\beta_1 d_2 + \beta_2 d_1\right) & B_2\beta_2 + B_3 d_2 & -B_2\beta_1 - B_3 d_1 & B_0 + B_1\left(-\beta_1^2 - \beta_2^2\right) \end{pmatrix}. \tag{S40}$$

For backwards propagation, many signs are flipped, producing

$$\mathbf{m}_\mathrm{B} = \begin{pmatrix} B_0 + B_1\left(d_1^2 + d_2^2\right) & -B_2 d_1 + B_3\beta_1 & B_2 d_2 - B_3\beta_2 & B_1\left(\beta_1 d_2 - \beta_2 d_1\right) \\ -B_2 d_1 + B_3\beta_1 & B_0 + B_1\left(-\beta_2^2 + d_1^2\right) & B_1\left(-\beta_1\beta_2 - d_1 d_2\right) & B_2\beta_2 + B_3 d_2 \\ B_2 d_2 - B_3\beta_2 & B_1\left(-\beta_1\beta_2 - d_1 d_2\right) & B_0 + B_1\left(-\beta_1^2 + d_2^2\right) & B_2\beta_1 + B_3 d_1 \\ -B_1\left(\beta_1 d_2 - \beta_2 d_1\right) & -B_2\beta_2 - B_3 d_2 & -B_2\beta_1 - B_3 d_1 & B_0 + B_1\left(-\beta_1^2 - \beta_2^2\right) \end{pmatrix}. \tag{S41}$$

The action of a mirror on incident light normal to it is to preserve the $x - y$ axes while flipping the $x' - y'$ and $r - l$ ones. In terms of the Mueller calculus,

$$\mathbf{m}_\mathrm{M} = \begin{pmatrix} 1 & 0 & 0 & 0 \\ 0 & 1 & 0 & 0 \\ 0 & 0 & -1 & 0 \\ 0 & 0 & 0 & -1 \end{pmatrix}. \tag{S42}$$

The matrix product $\mathbf{m}_\mathrm{M}\mathbf{m}_\mathrm{B}\mathbf{m}_\mathrm{M}$ represents the process of reflecting light off a mirror, propagating it backwards through a homogeneous sample, and then reflecting the light once again such that it propagates in the same direction as originally. Application of linear algebra demonstrates that

$$\mathbf{m}_\mathrm{M}\mathbf{m}_\mathrm{B}\mathbf{m}_\mathrm{M} = \begin{pmatrix} B_0 + B_1\left(d_1^2 + d_2^2\right) & -B_2 d_1 + B_3\beta_1 & -B_2 d_2 + B_3\beta_2 & -B_1\left(\beta_1 d_2 - \beta_2 d_1\right) \\ -B_2 d_1 + B_3\beta_1 & B_0 + B_1\left(-\beta_2^2 + d_1^2\right) & -B_1\left(-\beta_1\beta_2 - d_1 d_2\right) & -B_2\beta_2 - B_3 d_2 \\ -B_2 d_2 + B_3\beta_2 & -B_1\left(-\beta_1\beta_2 - d_1 d_2\right) & B_0 + B_1\left(-\beta_1^2 + d_2^2\right) & B_2\beta_1 + B_3 d_1 \\ B_1\left(\beta_1 d_2 - \beta_2 d_1\right) & B_2\beta_2 + B_3 d_2 & -B_2\beta_1 - B_3 d_1 & B_0 + B_1\left(-\beta_1^2 - \beta_2^2\right) \end{pmatrix}, \tag{S43}$$

which is equal to $\mathbf{m}_{\mathrm{F}}$, demonstrating that this combination of transformations is the same as that of forward propagation. That is, the entire cavity system may be considered as a continuous medium with reciprocal boundaries owing to its unique symmetries.

Supplementary Figure 1: Inversion of inter-dipole angle

If one inverts the inter-dipole angles, the sample is effectively flipped upon which its chiroptical behavior flips as well. Supplementary Fig. 1 compares 2D chiral polaritons for the minimal configuration of two transition dipoles under the angle β_{21} considered in the main text (45°) and its reverse (−45°).

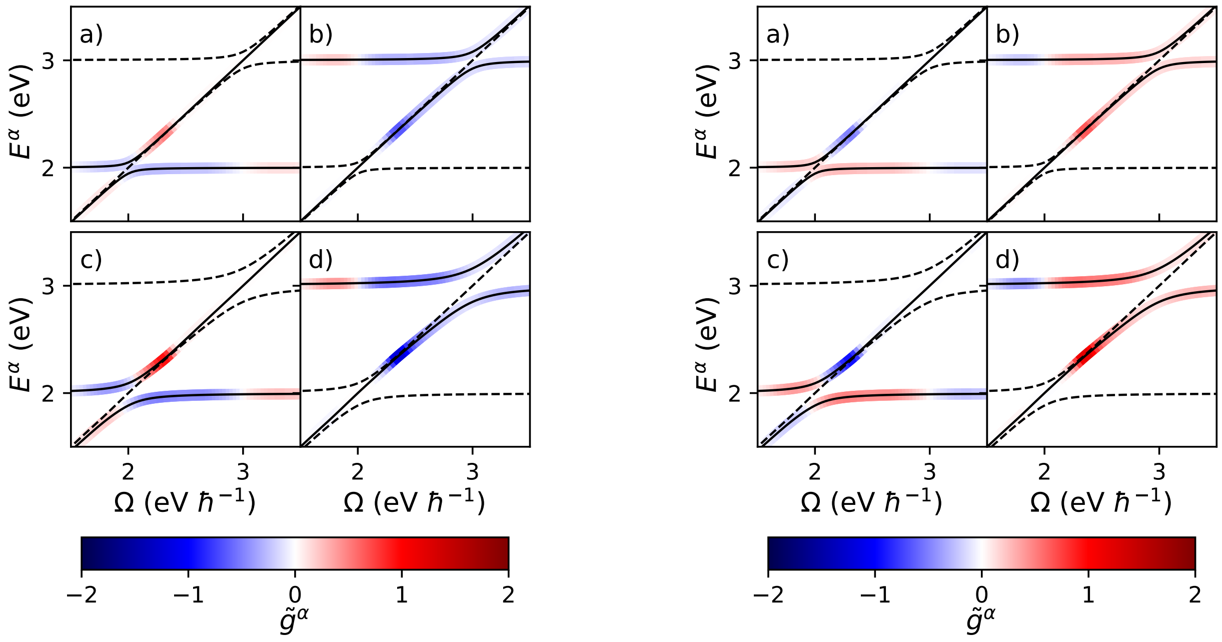

Supplementary Figure 1: Reproduction of Fig. 3 (left) and comparative results with reversed inter-dipole angle −45° (right). Subfigures analogous to those in main text.

Supplementary References

(1) Harris, C. R. et al. Array programming with NumPy. *Nature* **2020**, *585*, 357–362.

(2) Virtanen, P. et al. SciPy 1.0: Fundamental Algorithms for Scientific Computing in Python. *Nat. Methods* **2020**, *17*, 261–272.

(3) Hunter, J. D. Matplotlib: A 2D graphics environment. *Comput. Sci. Eng,* **2007**, *9*, 90–95.

(4) Berova, N.; Polavarapu, P. L.; Nakanishi, K.; Woody, R. W. *Comprehensive chiroptical spectroscopy Volume 1: Instrumentation, Methodologies, and Theoretical Simulations*; John Wiley & Sons, 2011; Vol. 1.

(5) Agranovich, V. M.; Ginzburg, V. *Crystal optics with spatial dispersion, and excitons*, 2nd ed.; Springer Science & Business Media, 2013; Vol. 42.

(6) Tavazzi, S.; Raimondo, L.; Silvestri, L.; Spearman, P.; Camposeo, A.; Polo, M.; Pisignano, D. Dielectric tensor of tetracene single crystals: The effect of anisotropy on polarized absorption and emission spectra. *J. Chem. Phys.* **2008**, *128*, 154709.

(7) Bassani, F.; Parravicini, G. P.; Ballinger, R. A. *Electronic states and optical transitions in solids*; Franklin Book Co., 1993; Vol. 1.

(8) Dresselhaus, M. S. Lecture notes in Solid State Physics Part II: Optical Properties of Solids. 2001.; `https://web.mit.edu/6.732/www/opt.pdf`.

(9) Duke, C.; Mahan, G. Phonon-broadened impurity spectra. I. Density of states. *Phys. Rev.* **1965**, *139*, 1965–1982.

(10) Zhu, Y.; Gauthier, D. J.; Morin, S.; Wu, Q.; Carmichael, H.; Mossberg, T. Vacuum Rabi splitting as a feature of linear-dispersion theory: Analysis and experimental observations. *Phys. Rev. Lett.* **1990**, *64*, 2499.

(11) Jensen, H.; Schellman, J.; Troxell, T. Modulation techniques in polarization spectroscopy. *Appl. Spectrosc.* **1978**, *32*, 192–200.

(12) Shindo, Y.; Nakagawa, M.; Ohmi, Y. On the Problems of CD Spectropolarimeters. II: Artifacts in CD Spectrometers. *Appl. Spectrosc.* **1985**, *39*, 860–868.

(13) Salij, A.; Goldsmith, R. H.; Tempelaar, R. Theory of Apparent Circular Dichroism Reveals the Origin of Inverted and Noninverted Chiroptical Response under Sample Flipping. *J. Am. Chem. Soc.* **2021**, *143*, 21519–21531.

(14) Brown, C. S.; Bak, A. E. General Lorentz transformation and its application to deriving and evaluating the Mueller matrices of polarization optics. Proc. SPIE 3754, Polarization: Measurement, Analysis, and Remote Sensing II. 1999; pp 65 – 74.

(15) Baranov, D. G.; Wersall, M.; Cuadra, J.; Antosiewicz, T. J.; Shegai, T. Novel nanostructures and materials for strong light–matter interactions. *ACS Photonics* **2018**, *5*, 24–42.

(16) Tavis, M.; Cummings, F. The exact solution of N two level systems interacting with a single mode, quantized radiation field. *Phys. Lett. A* **1967**, *25*, 714–715.
