# Supplementary figures and images for: Theory predicts 2D chiral polaritons based on achiral Fabry–Pérot cavities using apparent circular dichroism
Source: Nat Commun. 2024 Jan 6;15:340. doi: 10.1038/s41467-023-44523-1 (PMC10771534; doi:10.1038/s41467-023-44523-1)

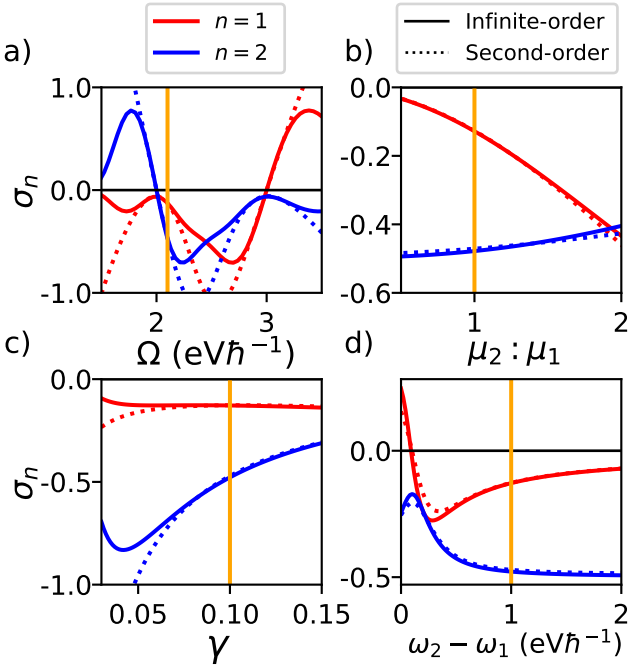

Supplement: Supplementary file 3 — Source Data [file 41467_2023_44523_MOESM3_ESM.zip › SourceFiles2DChiralPol/Figure 2/Figure 2.pdf]

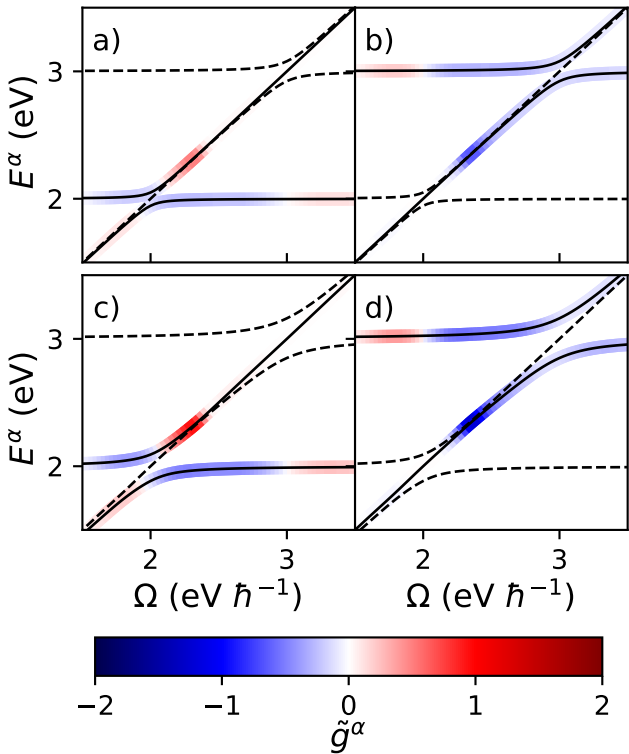

Supplement: Supplementary file 3 — Source Data [file 41467_2023_44523_MOESM3_ESM.zip › SourceFiles2DChiralPol/Figure 3/Figure 3.pdf]

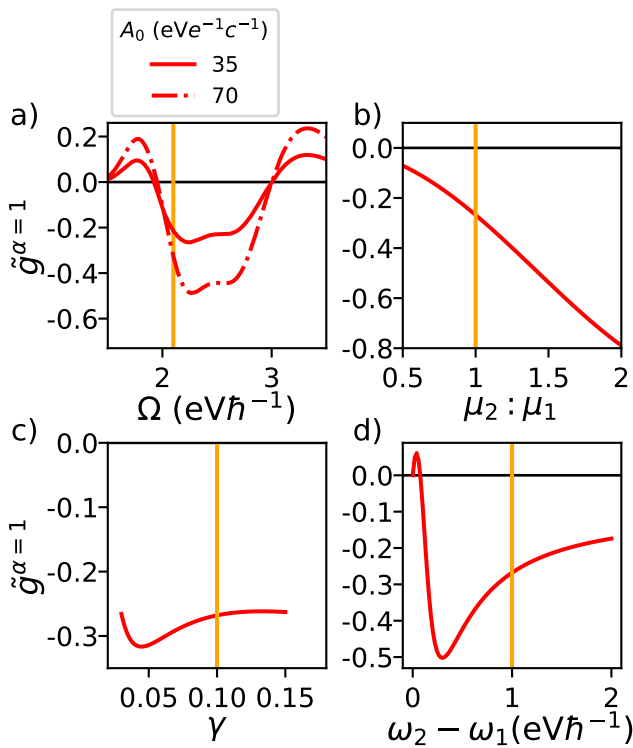

Supplement: Supplementary file 3 — Source Data [file 41467_2023_44523_MOESM3_ESM.zip › SourceFiles2DChiralPol/Figure 4/Figure 4.pdf]

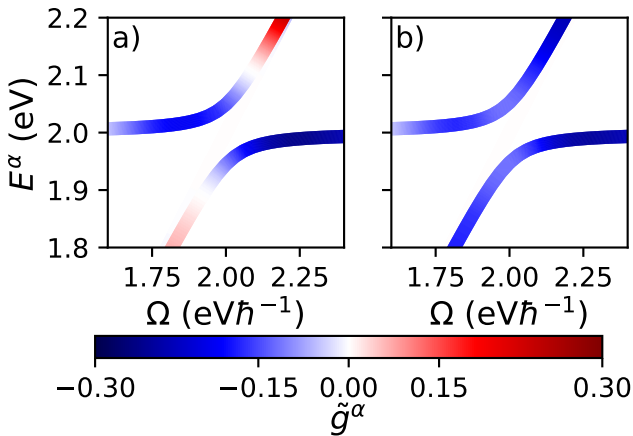

Supplement: Supplementary file 3 — Source Data [file 41467_2023_44523_MOESM3_ESM.zip › SourceFiles2DChiralPol/Figure 5/Figure 5.pdf]

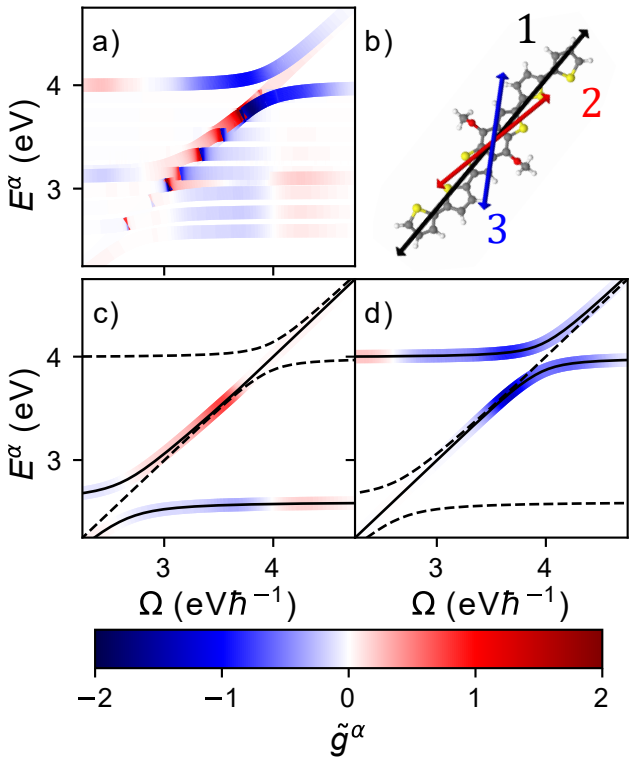

Supplement: Supplementary file 3 — Source Data [file 41467_2023_44523_MOESM3_ESM.zip › SourceFiles2DChiralPol/Figure 6/Figure 6.pdf]

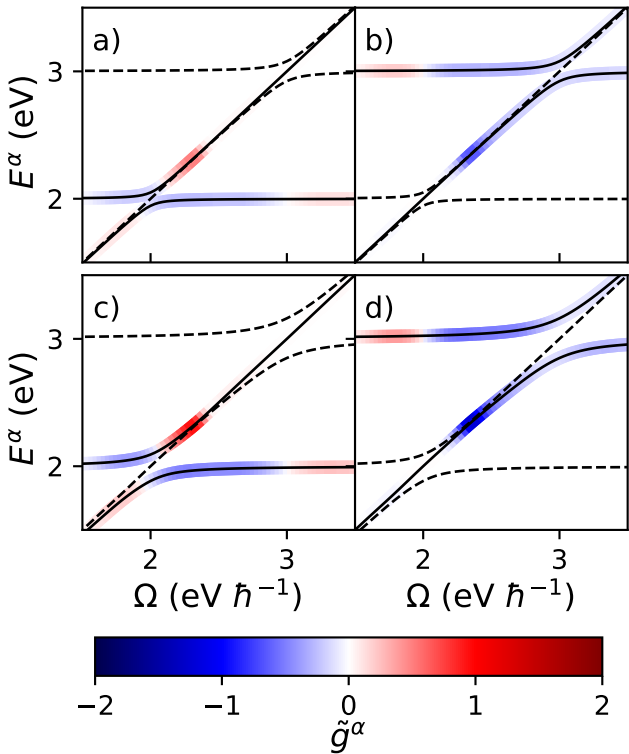

Supplement: Supplementary file 3 — Source Data [file 41467_2023_44523_MOESM3_ESM.zip › SourceFiles2DChiralPol/Supplementary Figure 1/Supplementary Figure 1 (left).pdf]

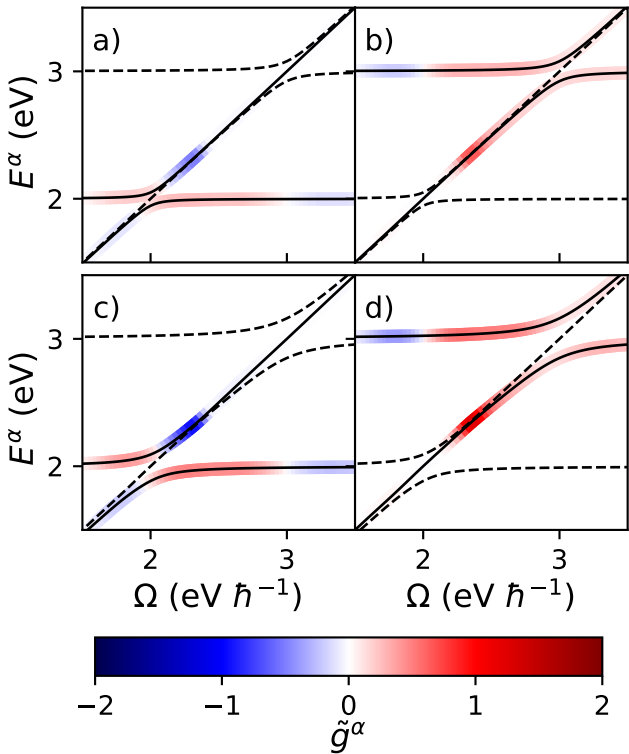

Supplement: Supplementary file 3 — Source Data [file 41467_2023_44523_MOESM3_ESM.zip › SourceFiles2DChiralPol/Supplementary Figure 1/Supplementary Figure 1 (right).pdf]
